# Inflammatory monocytes provide a niche for *Salmonella* expansion in the lumen of the inflamed intestine

**DOI:** 10.1371/journal.ppat.1007847

**Published:** 2019-07-15

**Authors:** Patrick A. McLaughlin, Julie A. Bettke, Jason W. Tam, Jesse Leeds, James B. Bliska, Brian P. Butler, Adrianus W. M. van der Velden

**Affiliations:** 1 Department of Molecular Genetics and Microbiology, Renaissance School of Medicine at Stony Brook University, Stony Brook, New York, United States of America; 2 Department of Pathobiology, School of Veterinary Medicine at Saint George’s University, Grenada, West-Indies; University of California Davis School of Medicine, UNITED STATES

## Abstract

*Salmonella* exploit host-derived nitrate for growth in the lumen of the inflamed intestine. The generation of host-derived nitrate is dependent on *Nos2*, which encodes inducible nitric oxide synthase (iNOS), an enzyme that catalyzes nitric oxide (NO) production. However, the cellular sources of iNOS and, therefore, NO-derived nitrate used by *Salmonella* for growth in the lumen of the inflamed intestine remain unidentified. Here, we show that iNOS-producing inflammatory monocytes infiltrate ceca of mice infected with *Salmonella*. In addition, we show that inactivation of type-three secretion system (T3SS)-1 and T3SS-2 renders *Salmonella* unable to induce CC- chemokine receptor-2- and CC-chemokine ligand-2-dependent inflammatory monocyte recruitment. Furthermore, we show that the severity of the pathology of *Salmonella*- induced colitis as well as the nitrate-dependent growth of *Salmonella* in the lumen of the inflamed intestine are reduced in mice that lack *Ccr2* and, therefore, inflammatory monocytes in the tissues. Thus, inflammatory monocytes provide a niche for *Salmonella* expansion in the lumen of the inflamed intestine.

## Author summary

*Salmonella* exploit gut inflammation to edge out competing microbes in the intestinal lumen and establish infection. Notably, *Salmonella* use inflammation-derived nitrate for growth in the intestinal lumen. The generation of inflammation-derived nitrate is dependent on *Nos2*, which encodes inducible nitric oxide synthase (iNOS), an enzyme that catalyzes nitric oxide production. However, the cellular sources of iNOS and, therefore, the nitric oxide-derived nitrate used by *Salmonella* for growth in the inflamed intestine remain unidentified, presenting an important, long-standing open question. Here, we show that iNOS-producing inflammatory monocytes, which are phagocyte precursors that play a critical role in immunity and host defense, promote nitrate-dependent *Salmonella* expansion in the inflamed intestine, providing new insights into how *Salmonella* exploit gut inflammation to establish infection.

## Introduction

*Salmonella enterica* serovars are pathogenic bacteria that cause significant morbidity and mortality in humans worldwide [1–4]. Non-typhoidal *Salmonella* (NTS) gastroenteritis is the most prevalent of the clinical syndromes associated with *Salmonella enterica* serovars. The global burden of NTS gastroenteritis is estimated to account for 93.8 million cases of disease annually, with 155,000 deaths [3].

*Salmonella enterica* serovars such as *Salmonella enterica* serovar Typhimurium (STm) invade intestinal epithelial cells and induce a secretory response in the intestinal epithelium that initiates the recruitment of innate immune cells [5]. This inflammatory response is characterized by dense, suppurative-to-pyogranulomatous infiltration of the lamina propria and submucosa, and multifocal pyogranulomatous lymphadenitis [6–8]. While this inflammatory response helps limit STm invasion in the long term, numerous studies have shown that STm exploit gut inflammation to edge out competing microbes in the intestinal lumen and establish infection [9, 10]. Specifically, the inflammatory response changes the composition of the gut microbiota and suppresses its growth [11–13]. Furthermore, STm use by-products of the inflammatory response such as nitrate and tetrathionate as electron acceptors for anaerobic respiration and growth in the lumen of the inflamed intestine [14, 15]. The resulting increase in relative abundance of STm, a facultative anaerobe, is accompanied by changes in the gut microbiota that are characterized by a marked decrease in relative abundance of obligate anaerobes (*e*.*g*., Clostridia) [12, 13, 16].

Host-derived nitrate is the preferred electron acceptor used by STm for anaerobic respiration and growth in the lumen of the inflamed intestine [17, 18]. The generation of host-derived nitrate is dependent on *Nos2* [19], which encodes inducible nitric oxide synthase (iNOS), an enzyme that catalyzes the production of nitric oxide (NO). However, the cellular source(s) of iNOS and, therefore, the NO-derived nitrate used by STm for growth in the lumen of the inflamed intestine remain unidentified.

Here, we show that iNOS-producing inflammatory monocytes (IM) infiltrate ceca of mice infected with STm and that inactivation of type-three secretion system (T3SS)-1 and T3SS-2 renders STm unable to induce CC-chemokine receptor-2 (CCR2)- and CC-chemokine ligand-2 (CCL2)-dependent IM recruitment. Furthermore, we provide evidence indicating that IM contribute to the severity of the pathology of STm-induced colitis and the generation of host-derived nitrate used by STm for growth in the lumen of the inflamed intestine. Collectively, these findings indicate that IM provide a niche for STm expansion in the lumen of the inflamed intestine.

## Results

### iNOS-producing IM infiltrate ceca of mice infected with STm

We previously showed that IM (*i*.*e*., CD11b^+^ Ly6C^hi^ Ly6G^-^ cells) recruited from bone marrow into tissues of mice persistently infected with STm produce NO by an iNOS-dependent mechanism [20]. To begin to evaluate the role of IM in NTS gastroenteritis, we used the well-established C57BL/6J mouse model of STm-induced colitis [6]. This widely used model is highly relevant because the robust inflammatory response elicited during STm infection closely resembles the pathology of NTS gastroenteritis in humans [7, 8]. The model involves treatment of the mice with a single dose of streptomycin (Sm) prior to STm infection [6], which lowers colonization resistance and induces dysbiosis of the gut microbiota [13]. Without Sm pretreatment, STm does not induce the robust inflammatory response that resembles the pathology of NTS gastroenteritis in humans [6].

**Fig 1:**
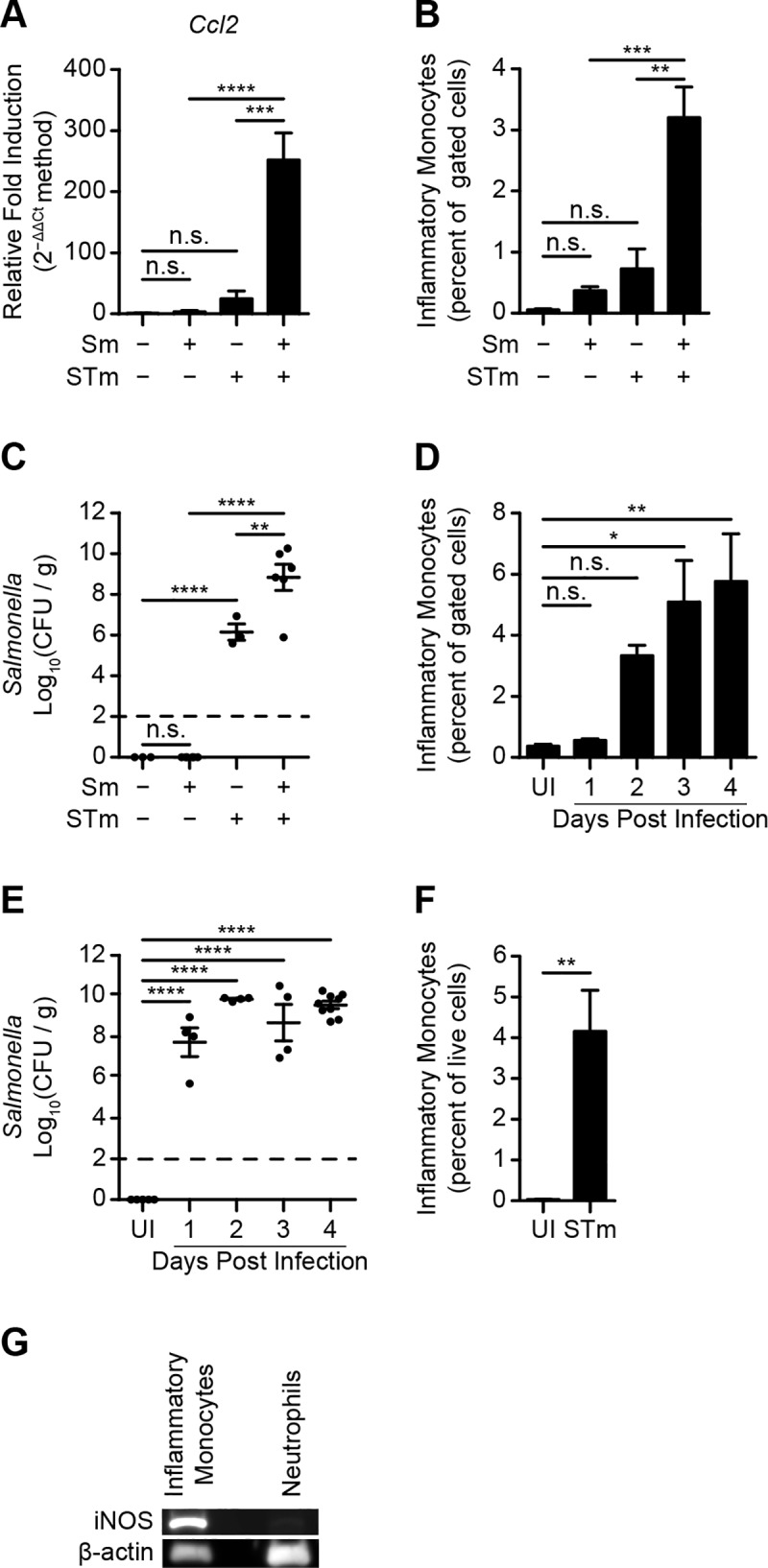
iNOS-producing IM infiltrate ceca of mice infected with STm. (A-C) Effects of Sm pretreatment and STm infection on C57BL/6J mice (n = 3–7 per group). Samples were harvested on day 4 after infection. Data show mean with SEM (A and B) or mean with SEM and individual data points (C), and were analyzed by one-way ANOVA with Sidak post hoc test. (A) Relative fold induction of *Ccl2* expression in cecal tissues, normalized to *Gapdh*. (B) Percentage of IM among total gated cells from cecal tissues. (C) Number of STm CFU recovered from cecal contents, normalized to weight. (D-E) Four-day time course tracking IM recruitment into cecal tissues from C57BL/6J mice (n = 4–9 per group) treated with Sm prior to inoculation with STm. Mice treated with Sm prior to inoculation with PBS (uninfected, UI) were used as a control. Data show mean with SEM (D) or mean with SEM and individual data points (E), and were analyzed by one-way ANOVA with Fisher’s LSD post hoc test. (D) Percentage of IM among total gated cells from cecal tissues. (E) Number of STm CFU recovered from cecal contents, normalized to weight. (F) Percentage of IM among live cells in cecal contents from C57BL/6J mice (n = 6 per group) treated with Sm prior to inoculation with PBS (uninfected, UI) or STm. Cecal contents were collected on day 4 after inoculation. Data show mean with SEM and were analyzed by Student’s *t*-test. (G) Level of iNOS expressed by IM and neutrophils purified and pooled from cecal tissues of C57BL/6J mice (n = 5–6 per group) treated with Sm prior to inoculation with STm. Ceca were harvested on day 4 after inoculation. β-Actin was used as a loading control for Western blotting. Data are representative of two independent experiments. Also see S1 Fig.

C57BL/6J mice were left untreated or treated with Sm one day prior to inoculation with PBS or STm (suspended in PBS). Transcript analysis revealed that, by day 4 after inoculation, expression of *Ccl2*, which encodes chemokine (C-C motif) ligand-2 (CCL2), a potent monocyte chemoattractant, had been induced in ceca of mice pretreated with Sm and inoculated with STm, but not in ceca of mice left untreated or treated with Sm or STm alone (**Fig 1A**). Consistent with this finding, flow cytometric analysis revealed that IM had accumulated in ceca of mice pretreated with Sm and inoculated with STm, but not in ceca of mice left untreated or treated with Sm or STm alone (**Fig 1B and S1A Fig**). As expected, similar results were obtained with respect to neutrophilic granulocyte recruitment (**S1A and S1B Fig**). Furthermore, bacterial burden assays showed that, at the time of harvest, STm were present in the cecal lumen of all mice inoculated with STm, with the number of STm colony forming units (CFU) recovered being greatest in mice treated with Sm (**Fig 1C**).

Follow-up experiments revealed that both IM (**Fig 1D**) and neutrophilic granulocytes (*i*.*e*., CD11b^+^ Ly6C^int^ Ly6G^+^ cells) (**S1C Fig**) accumulated in ceca of infected mice (**Fig 1E**) over time, and were also found in the cecal lumen (**Fig 1F and S1D Fig**). Furthermore, Western blot analysis revealed that IM but not neutrophilic granulocytes purified by FACS from ceca of Sm-pretreated mice inoculated with STm expressed iNOS (**Fig 1G**). Thus, iNOS-producing IM infiltrate ceca of mice infected with STm.

### Inactivation of T3SS-1 and T3SS-2 renders STm unable to induce *Ccr2*- and *Ccl2*-dependent IM recruitment

STm causes intestinal inflammation by employing two type-three secretion systems, T3SS-1 and T3SS-2, which mediate epithelial cell invasion and intramacrophage survival, respectively [21, 22]. Inactivation of T3SS-1 (through a mutation in *invA*, which encodes an essential structural component of T3SS-1 [21]) and T3SS-2 (through a mutation in *ssaD*, which encodes an essential structural component of T3SS-2 [21]) renders STm unable to induce intestinal inflammation in the murine model of STm-induced colitis [23]. Interestingly, mutant strains of STm that do not induce colitis or cannot respire nitrate exhibit a reduced ability to compete with other microbes in the lumen of the gut [13–15, 24].

**Fig 2:**
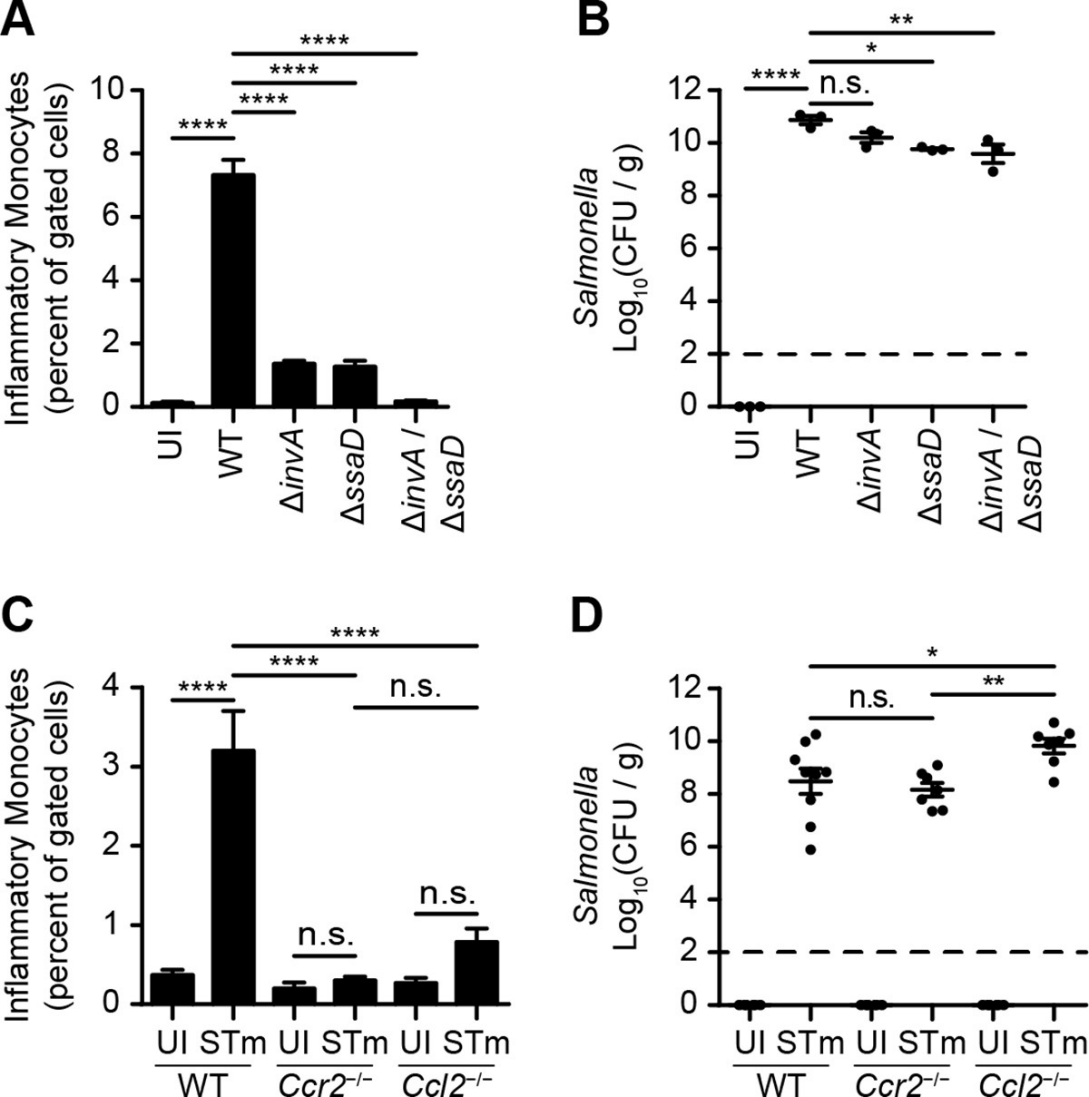
Inactivation of T3SS-1 and T3SS-2 renders STm unable to induce *Ccr2*- and *Ccl2*-dependent IM recruitment. (A) Percentage of IM among total gated cells in cecal tissues from C57BL/6J mice (n = 3 per group) treated with Sm prior to inoculation with PBS (uninfected; UI) or wild-type (WT) STm, or *invA* (T3SS-1), *ssaD* (T3SS-2), or *invA ssaD* (T3SS-1 T3SS-2) mutant STm. Ceca were harvested on day 4 after inoculation. Data show mean with SEM and were analyzed by one-way ANOVA with Sidak post hoc test. (B) Corresponding number of STm CFU recovered from cecal contents, normalized to weight. Data show mean with SEM and individual data points, and were analyzed by one-way ANOVA with Sidak post hoc test. (C) Percentage of IM among total gated cells in cecal tissues from C57BL/6J (WT), *Ccr2*^-/-^, and *Ccl2*^-/-^ mice (n = 6–9 per group) treated with Sm prior to inoculation with PBS (uninfected, UI) or STm. Ceca were harvested on day 4 after inoculation. Data show mean with SEM and were analyzed by one-way ANOVA with Sidak post hoc test. (D) Corresponding number of STm CFU recovered from cecal contents, normalized to weight. Data show mean with SEM and individual data points, and were analyzed by one-way ANOVA with Sidak post hoc test. Also see S2 Fig.

To determine if T3SS-1 and T3SS-2 are required for STm to induce IM infiltration of the cecum, mice were treated with Sm prior to intragastric inoculation with PBS, wild-type (WT) STm, or *invA*, *ssaD*, or *invA ssaD* mutant STm. On day 4 after inoculation, IM had accumulated in ceca of mice inoculated with WT STm, but not *invA*, *ssaD*, or *invA ssaD* mutant STm, with accumulation being lowest in mice inoculated with *invA ssaD* mutant STm (**Fig 2A**). Similar results were obtained with respect to neutrophilic granulocyte recruitment (**S2A Fig**). As expected, lower numbers of *invA*, *ssaD*, and *invA ssaD* mutant STm (relative to WT STm) were recovered from the cecal lumen of infected mice, with numbers of *invA ssaD* mutant STm being lowest (**Fig 2B**).

CCR2 plays a critical role in the emigration of Ly6C^hi^ monocytes from bone marrow [25], while CCL2 is a known ligand for CCR2 [26]. To evaluate the roles of CCR2 and CCL2 in STm-induced colitis, WT, *Ccr2*-deficient (*Ccr2*^-/-^), and *Ccl2*-deficient (*Ccl2*^-/-^) mice in the C57BL/6J strain background were treated with Sm prior to intragastric inoculation with PBS or STm. On day 4 after inoculation, IM had infiltrated ceca of infected WT but not *Ccr2*^-/-^ or *Ccl2*^-/-^ mice, with substantial numbers of STm recovered from the cecal lumen (**Fig 2C and 2D**). As expected, neutrophilic granulocytes had infiltrated ceca of infected WT, *Ccr2*^-/-^, and *Ccl2*^-/-^ mice (**S2B Fig**). Collectively, these results indicate that inactivation of T3SS-1 and T3SS-2 renders STm unable to induce *Ccr2*- and *Ccl2*-dependent IM recruitment.

### IM contribute to the severity of the pathology of STm-induced colitis

**Fig 3:**
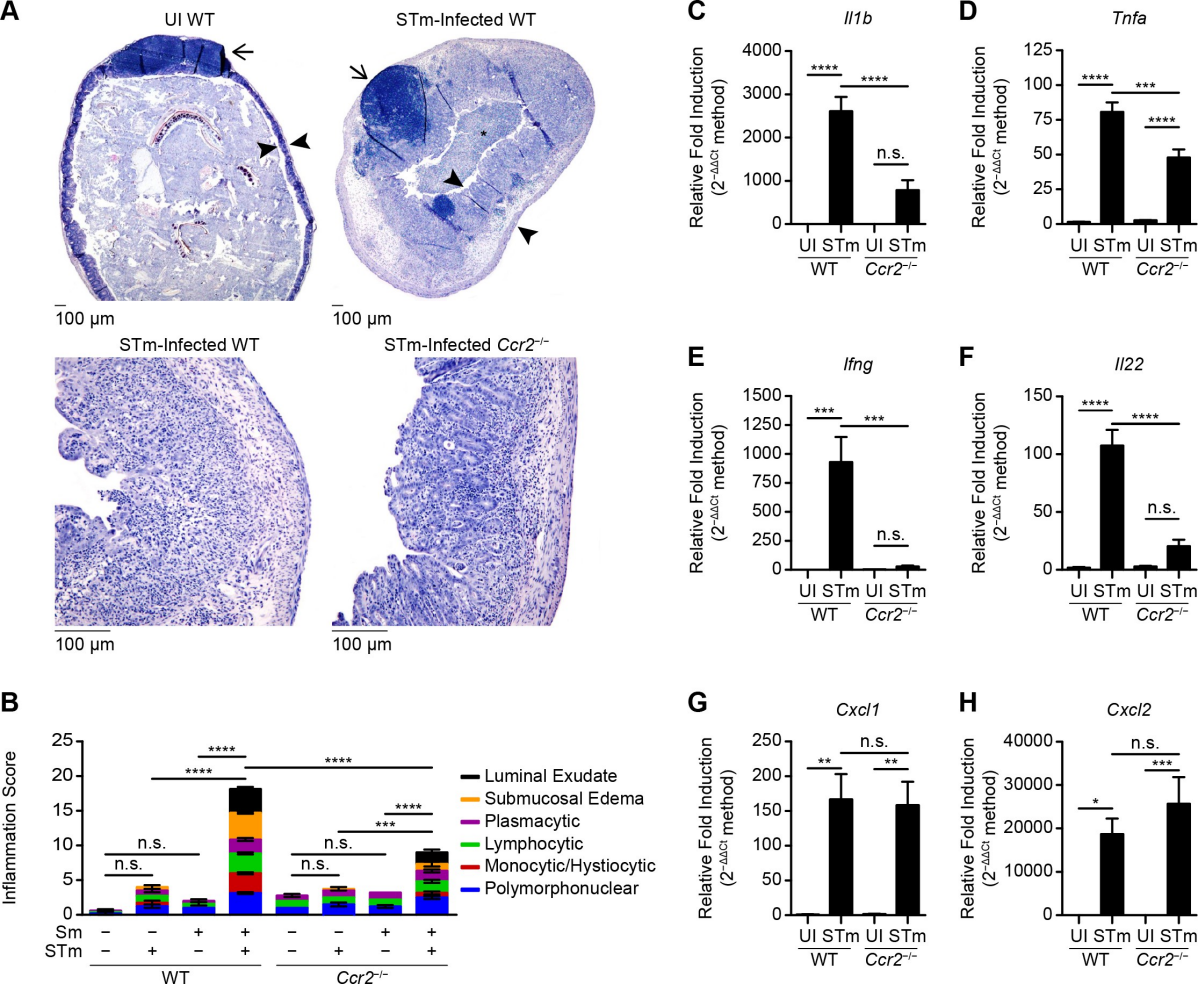
IM contribute to the severity of the pathology of STm-induced colitis. (A) Representative hematoxylin- and eosin-stained cecal sections from C57BL/6J (WT) and *Ccr2*^-/-^ mice (n = 5–7 per group) treated with Sm prior to inoculation with PBS or STm. Ceca were harvested on day 4 after inoculation. Top left: WT mouse treated with Sm prior to inoculation with PBS. Top right: WT mouse treated with Sm prior to inoculation with STm. Bottom left: WT mouse treated with Sm prior to inoculation with STm. Bottom right: *Ccr2*^-/-^ mouse treated with Sm prior to inoculation with STm. Arrows point to cecal lymphoid tissue. Arrowheads delineate the cecal wall. Asterisk indicates accumulation of inflammatory exudate within the cecal lumen. (B) Blinded, semi-quantitative histopathological analysis of cecal tissues from C57BL/6J (WT) and *Ccr2*^-/-^ mice (n = 4–7 per group) left untreated or treated with Sm prior to inoculation with PBS or STm. Ceca were harvested on day 4 after inoculation. Data show mean with SEM and were analyzed by one-way ANOVA with Sidak post hoc test. (C-H) Relative fold induction of *Il1b* (C), *Tnfa* (D), *Ifng* (E), *Il22* (F), *Cxcl1* (G), and *Cxcl2* (H) expression in cecal tissues from C57BL/6J (WT) and *Ccr2*^-/-^ mice (n = 5–7 per group) treated with Sm prior to inoculation with PBS (uninfected, UI) or STm. Ceca were harvested on day 4 after inoculation. Relative target gene expression was normalized to *Gapdh*. Data show mean with SEM and were analyzed by one-way ANOVA with Sidak post hoc test.

To evaluate the contribution of IM to the pathology of STm-induced colitis, WT and *Ccr2*^-/-^ mice were left untreated or treated with Sm prior to intragastric inoculation with PBS or STm. On day 4 after inoculation, ceca were harvested and processed for blinded, semiquantitative histopathological analysis. As expected, the robust inflammatory response elicited in ceca of WT mice treated with Sm and inoculated with STm (relative to WT mice left untreated or WT mice treated with Sm or STm alone) was characterized by dense suppurative-to-pyogranulomatous infiltration of the lamina propria and submucosa (**Fig 3A and 3B**). Additional histopathological changes associated with STm infection included erosion and attenuation of the surface epithelium, crypt hyperplasia and abscessation, lymphocytolysis and lymphadenitis of the cecal lymphoid tissue, and accumulation of suppurative exudate in the cecal lumen (**Fig 3A and 3B**). In contrast, the inflammatory response elicited in ceca of *Ccr2*^-/-^ mice treated with Sm and inoculated with STm (relative to *Ccr2*^-/-^ mice left untreated or *Ccr2*^-/-^ mice treated with Sm or STm alone) was significantly less robust (**Fig 3A and 3B**). Furthermore, ceca from *Ccr2*^-/-^ mice treated with Sm and inoculated with STm displayed an overall inflammation score that was attenuated (relative to ceca from WT mice treated with Sm and inoculated with STm) and indicative of an inflammatory infiltrate characterized as predominantly neutrophilic, with appreciable attenuation of mononuclear cell recruitment (**Fig 3B**).

In parallel experiments, transcript analysis revealed that STm-induced expression of *Il1b*, *Tnfa*, *Ifng*, and *Il22*, which encode the proinflammatory cytokines interleukin-1β, tumor necrosis factor-β, interferon-γ, and interleukin-22, respectively, was diminished in Sm-pretreated *Ccr2*^-/-^ mice relative to WT mice (**Fig 3C–3F**). In contrast, STm-induced expression of *Cxcl1* and *Cxcl2*, which encode neutrophil chemoattractants chemokine (C-X-C motif) ligand-1 and -2, respectively, was normal in Sm-pretreated *Ccr2*^-/-^ mice relative to WT mice (**Fig 3G and 3H**). Collectively, these results indicate that IM contribute to the severity of the pathology of STm-induced colitis.

### IM contribute to the STm-induced generation of nitrate in the cecal mucosa

**Fig 4:**
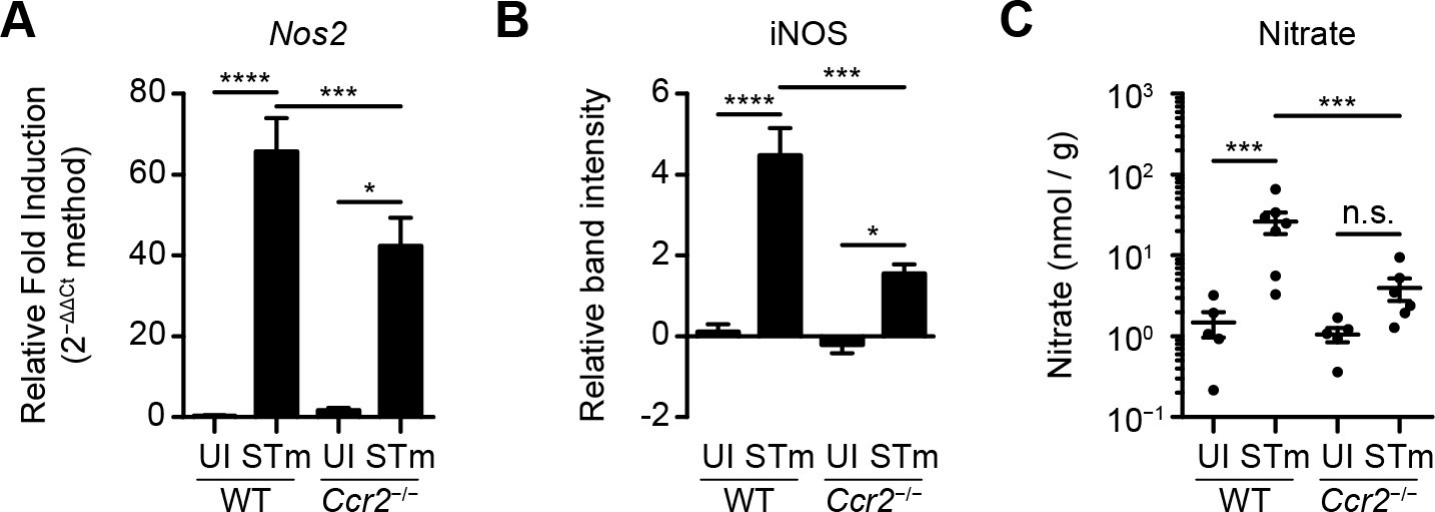
IM contribute to the STm-induced generation of nitrate in the cecal mucosa. (A) Relative fold induction of *Nos2* expression in cecal tissues from C57BL/6J (WT) and *Ccr2*^-/-^ mice (n = 5–7 per group) treated with Sm prior to inoculation with PBS (uninfected, UI) or STm. Ceca were harvested on day 4 after inoculation. Relative *Nos2* expression was normalized to *Gapdh*. Data show mean with SEM and were analyzed by one-way ANOVA with Sidak post hoc test. (B) Relative level of iNOS expressed in cecal tissues from C57BL/6J (WT) and *Ccr2*^-/-^ mice (n = 6–9 per group) treated with Sm prior to inoculation with PBS (uninfected, UI) or STm. Ceca were harvested on day 4 after inoculation. β-Actin was used as a loading control for Western blotting. Data show mean with SEM and were analyzed by one-way ANOVA with Sidak post hoc test. (C) Concentration of nitrate in cecal mucus from C57BL/6J (WT) and *Ccr2*^-/-^ mice (n = 5–7 per group) left untreated or treated with Sm prior to inoculation with PBS or STm. Ceca were harvested on day 4 after inoculation. Data show mean with SEM and individual data points, and were analyzed by one-way ANOVA with Sidak post hoc test. Also see S3 Fig.

To evaluate the contribution of CCR2 and, therefore, IM to STm-induced *Nos2* expression in the cecum, WT and *Ccr2*^-/-^ mice were treated with Sm prior to intragastric inoculation with PBS or STm. On day 4 after inoculation, ceca were harvested and total RNA was isolated from cecal tissues. Subsequent transcript analysis revealed that STm-induced *Nos2* expression in the cecum was diminished in *Ccr2*^-/-^ mice relative to WT mice, but not abolished (**Fig 4A**). Similar results were obtained when total protein was isolated from said cecal tissues and subjected to Western blot analysis to measure levels of iNOS (**Fig 4B and S3A Fig**).

In parallel experiments, a fluorometric 2,3-diaminonaphtalene (DAN) assay [27] was used to measure nitrate concentrations in cell-free cecal mucus extracts. Analysis of DAN assay results revealed that nitrate concentrations in cecal mucus from *Ccr2*^-/-^ mice treated with Sm and inoculated with STm were significantly lower than those in cecal mucus from WT mice treated with Sm and inoculated with STm (**Fig 4C**). Analysis of DAN assay results also revealed that, under these conditions, nitrate concentrations in cecal mucus from WT and *Ccr2*^-/-^ mice treated with Sm but left uninfected were comparable (**Fig 4C and S3B Fig**). Collectively, these results indicate that IM contribute to the STm-induced generation of nitrate in the cecal mucosa. The observed effects and effect sizes in *Ccr2*^-/-^ mice further suggest that there may be cells other than IM that also contribute to the STm-induced generation of nitrate in the cecal mucosa.

### IM contribute to the generation of host-derived nitrate used by STm for growth in the lumen of the inflamed intestine

**Fig 5:**
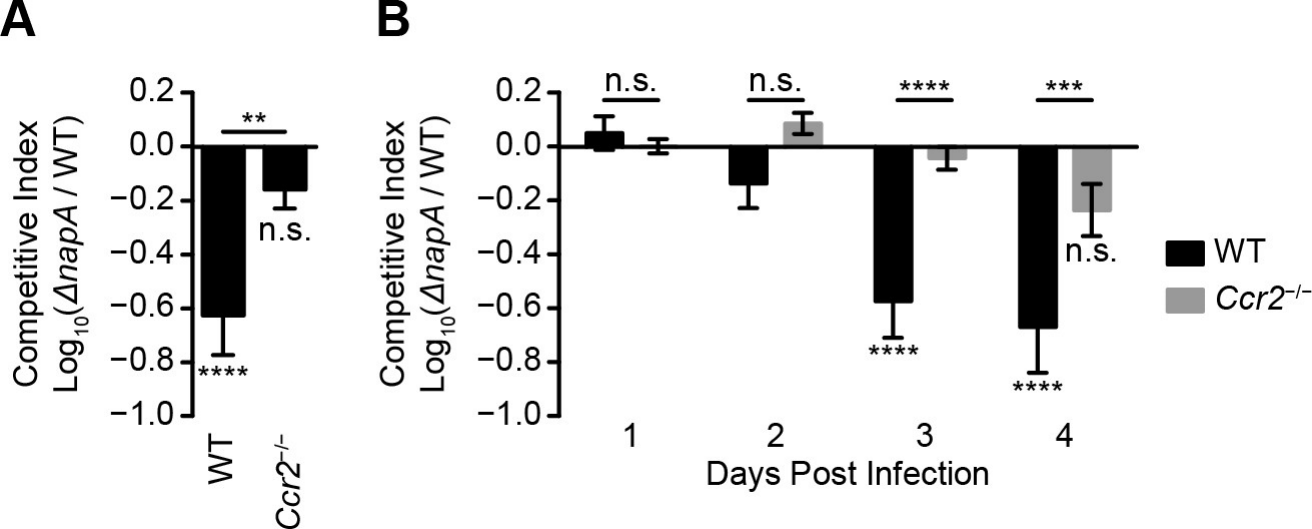
IM contribute to the generation of host-derived nitrate used by STm for growth in the lumen of the inflamed intestine. (A-B) Competitive indices of WT and *napA* mutant STm CFU in cecal contents (A) and fecal pellets (B) from C57BL/6J (WT) and *Ccr2*^-/-^ mice (n = 16 per group) treated with Sm prior to inoculation with a 1:1 mixture of differentially marked WT and *napA* mutant STm. Cecal contents were collected on day 4 after inoculation. Fecal pellets were collected daily up to day 4 after inoculation. Competitive indices were calculated by dividing the number of *napA* mutant STm CFU by the number of WT STm CFU, and normalized to the input ratio. Data show mean with SEM from three independent experiments and were analyzed by one-way ANOVA (A) or two-way ANOVA (B) with Sidak post hoc test.

To further evaluate the significance of these findings as they relate to the ability of STm to use host-derived nitrate for growth in the lumen of the inflamed intestine, WT and *Ccr2*^-/-^ mice were treated with Sm prior to intragastric inoculation with a 1:1 mixture of differentially marked WT and nitrate respiration-deficient *napA* mutant STm. Consistent with published results [14], WT STm had outcompeted *napA* mutant STm for growth in the cecal lumen of WT mice by day 4 after inoculation (**Fig 5A**). In contrast, however, WT STm had failed to outcompete *napA* mutant STm for growth in the cecal lumen of *Ccr2*^-/-^ mice (**Fig 5A**). Similar results were obtained when fecal samples were analyzed over time (**Fig 5B**). Collectively, these results indicate that IM contribute to the generation of host-derived nitrate used by STm for growth in the lumen of the inflamed intestine.

## Discussion

We report that iNOS-producing IM infiltrate ceca of mice infected with STm and that inactivation of T3SS-1 and T3SS-2 renders STm unable to induce *Ccr2*- and *Ccl2*-dependent IM recruitment. Furthermore, we report that IM contribute to the severity of the pathology of STm-induced colitis and the generation of host-derived nitrate used by STm for growth in the lumen of the inflamed intestine. Collectively, our findings indicate that IM promote post-antibiotic STm expansion in the lumen of the inflamed intestine, providing new, fundamental insights into how STm exploit gut inflammation to promote disease progression.

**Fig 6:**
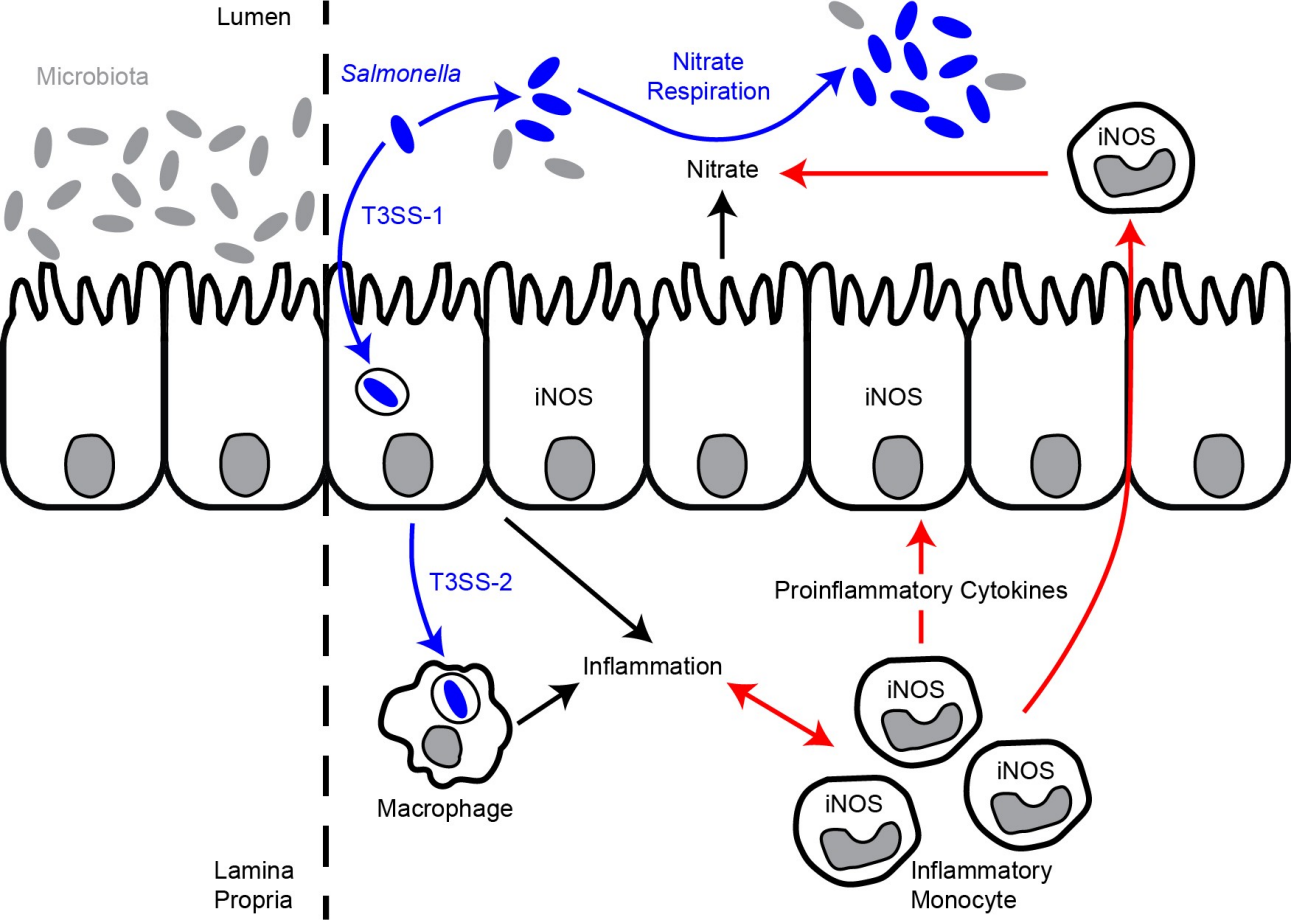
Model of how IM promote nitrate-dependent STm expansion in the lumen of the inflamed intestine. We postulate that IM contribute both directly and indirectly to the generation of host- derived nitrate used by STm for growth in the lumen of the inflamed intestine. IM may contribute directly to the generation of host-derived nitrate used by STm for growth in the lumen of the inflamed intestine by expressing *Nos2* and indirectly by potentiating *Nos2* expression by other cells such as IEC. See the text for details.

We postulate that IM contribute both directly and indirectly to the generation of host-derived nitrate used by STm for growth in the lumen of the inflamed intestine (**Fig 6**). IM may contribute directly to the generation of host-derived nitrate used by STm for growth in the lumen of the inflamed intestine by expressing *Nos2* (Fig 1G) and indirectly by potentiating *Nos2* expression by other cells such as intestinal epithelial cells (IEC) (**Fig 6**). Our model is consistent with the notion that IM may not be the sole source of host-derived nitrate and that cells other than IM (*e*.*g*., tissue-resident cells) may help promote nitrate-dependent STm expansion in the lumen of the inflamed intestine.

Epithelial *Nos2* expression is induced during the course of intestinal inflammation and in response to stimulation with various cytokines (*e*.*g*., IFN-γ, IL-22) [28–31]. Whether epithelial *Nos2* expression contributes to post-antibiotic STm expansion in the lumen of the inflamed intestine is not known. However, depletion of butyrate-producing gut microbes by antibiotic treatment reduces epithelial signaling through the intracellular butyrate sensor peroxisome proliferator-activated receptor (PPAR)-γ, resulting in increased epithelial *Nos2* expression, luminal nitrate availability, and dysbiotic Enterobacteriaceae expansion [32]. Thus, host-derived nitrate used by STm for growth in the lumen of the inflamed intestine may be generated by both IM and IEC, where the contribution of IM may be direct and indirect, and the contribution of IEC may be mediated by the depletion of butyrate-producing gut microbes and potentiated by proinflammatory cytokines.

Additionally, we postulate that IM contribute both directly and indirectly to the pathology of STm-induced colitis. IM may contribute directly to the pathology of STm-induced colitis by producing proinflammatory cytokines (*e*.*g*., TNF-α) and indirectly by licensing other cells (*e*.*g*., NK cells, γ:δ T cells, intraepithelial lymphocytes, group 3 innate lymphocytes) to produce proinflammatory cytokines (*e*.*g*., TNF-α, IFN-γ, IL-22), some of which, as stated above, can potentiate epithelial *Nos2* expression. Thus, IM may play a key role in coordinating induced innate immune responses in the inflamed intestine.

As for our finding that high bacterial burdens persist in the cecal lumen of mice infected with *invA ssaD* mutant *Salmonella* (Fig 2B), it is well established that *Salmonella* mutants unable to trigger gut inflammation can replicate to near wild-type levels in the non-inflamed intestine. Together with the notion that *Salmonella*, at least initially, replicates in a non-inflamed gut, our finding suggests that *Salmonella* can leverage multiple metabolic pathways to access diverse and redundant resources, including carbon and respiration substrates, from diverse origins (*e*.*g*., dietary products, host- derived products, and resident microbiota byproducts).

In summary, this work establishes that IM play a key role in the pathogenesis of STm-induced colitis. The conceptual advances resulting from this work are of broad relevance for understanding both infectious and digestive diseases such as NTS gastroenteritis, ulcerative colitis, and Crohn’s disease, all of which are characterized by intestinal inflammation and dysbiosis of the gut microbiota.

## Materials and methods

### Contact for reagent and resource sharing

Further information and requests for resources and reagents should be directed to and will be fulfilled by the Corresponding Author, Adrianus W.M. van der Velden (a.vandervelden@stonybrook.edu).

### Ethics statement

Studies that used live mice were performed in accordance with relevant institutional and national guidelines and regulations [33]. All experiments involving mice were approved by the Stony Brook University Institutional Animal Care and Use Committee (protocol number 2012-2075-11.12.21-FAR-Mi) and conform to the relevant regulatory standards. Euthanasia was conducted using carbon dioxide inhalation in accordance with the American Veterinary Medical Association Guidelines for Euthanasia of Animals (2013 Report of the AVMA Panel of Euthanasia). The Stony Brook University Animal Care and Use program operates in accordance with the US Department of Agriculture Animal Welfare Act (1966), Regulation (CFR, 2009) and policies; the Health Research Extension Act (1985), the Public Health Service Policy on Humane Care and Use of Laboratory Animals (PHS, 2002), the Guide for the Care and Use of Laboratory Animals (NRC, 2011), the New York State Law (Chapter II: Administrative Rules and Regulations, Chapter II, part 55 State Sanitary Code 16), and other applicable federal, state, and local laws, regulations, policies, and guidelines.

### Mice

Studies used commercially available C57BL/6J (The Jackson Laboratory Cat#000664; RRID: IMSR_JAX:000664), B6.129S4-*Ccr2*^*tm1lfc*^/J (*Ccr2*^-/-^; The Jackson Laboratory Cat#004999; RRID: IMSR_JAX004999), and B6.129S4-*Ccl2*^*tm1Rol*^/J (*Ccl2*^-/-^; The Jackson Laboratory Cat#004434; RRID IMSR_JAX004434) mice that were either purchased or bred in our specific pathogen-free animal facility at Stony Brook University. Mouse genotyping was performed by use of established protocols from The Jackson Laboratory. Studies used both male and female mice aged 8–20 weeks, sex- and age-matched by group. All mice were housed in the Division of Laboratory Animal Resources at Stony Brook University. The animal facility at Stony Brook University is accredited by AAALAC and licensed by the USDA and the State of New York Department of Health. Euthanasia of mice was performed by inhalation of carbon dioxide, a method that is consistent with the recommendations of the AVMA Panel on Euthanasia.

### Microbe strains

Studies used various strains of STm. STm strain IR715 [34], a spontaneous nalidixic acid-resistant derivative of STm strain 14028 (American Type Culture Collection), was used as the WT strain, except for studies involving mixed infections, where STm strain SL1344 [35] was used as the WT strain.

To generate an *invA* mutant STm strain in the IR715 strain background, bacteriophage P22 HT*int*-mediated transduction was used to move the *invA* mutation from an isogenic derivative of STm strain BA715 [36] that was generated when bacteriophage KB1*int*-mediated transduction was used to move the *invA* mutation from STm strain SWL2020 [37]. An isogenic derivative of STm strain IR715 lacking *ssaD* was generated by use of the lambda red recombinase method [38]. Briefly, the kanamycin resistance gene carried by plasmid pCLF4 [39] was amplified by use of PCR, with primers ssaD_mut Fwd and ssaD_mut Rev (**S1 Table**). The resulting PCR product was electroporated into STm strain IR715 carrying plasmid pKD46 [38] to ultimately generate, by homologous recombination, a kanamycin-resistant and ampicillin-sensitive *ssaD* mutant STm strain. This strain was verified by PCR and DNA sequencing after which bacteriophage P22 HT*int*-mediated transduction was used to move the *ssaD* mutation into STm strain IR715. To generate an *invA ssaD* mutant STm strain in the IR715 strain background, bacteriophage P22 HT*int*-mediated transduction was used to move the *ssaD* mutation from the *ssaD* mutant STm strain into the *invA* mutant STm strain. An isogenic derivative of STm strain SL1344 lacking *napA* was also generated by use of the lambda red recombinase method, essentially as described above and with primers napA_mut Fwd and napA_mut Rev (**S1 Table**).

With the use of standard microbiological techniques, bacteria were grown aerobically overnight at 37°C in lysogeny broth (3 ml) or on lysogeny agar. When necessary, carbenicillin (100 μg/ml; Teknova Cat#2105; CAS#4800-94-6), chloramphenicol (30 μg/ml; Sigma Cat#0378-25G; CAS#56-75-7), kanamycin (60 μg/ml; TCI America Cat#K0047-25G; CAS#25389-94-0), nalidixic acid (50 μg/ml; Alfa Aesar Cat#J63550-14; CAS#3374-05-8), or streptomycin (100 μg/ml; Fisher Scientific Cat#BP910-50; CAS#3810-74-0) was added to the culture medium to select for various (mutant) STm strains in the IR715 or SL1344 strain backgrounds.

### Mouse infections

Naïve mice were left untreated or treated with Sm (0.1 ml of a 200 mg/ml solution in PBS administered orally) one day prior to intragastric inoculation with 0.1 ml of PBS or 1 x 10^8^ CFU of STm suspended in PBS. To improve the consistency of the infections, food (but not water) was withheld for 6–8 hours prior to inoculation. Immediately after inoculation, food was provided *ad libitum*. Tenfold serial dilutions of the inoculum were plated to confirm the inoculum titer. At various times after inoculation (up to day 4), ceca were harvested and processed for downstream analysis, and cecal contents were collected for enumerating STm CFU.

For mixed infections, mice were treated with Sm prior to intragastric inoculation with 1 x 10^8^ CFU of a 1:1 mixture of differentially marked WT and *napA* mutant STm in the SL1344 strain background (to assess the ability of the host to generate nitrate used by STm for growth in the lumen of the gut). WT and *napA* mutant STm in the SL1344 strain background were used because, unlike STm strain IR715, STm strain SL1344 carries a prophage encoding SopE, a type III effector that boosts nitrate-dependent STm expansion in the lumen of the inflamed intestine by enhancing iNOS expression in the cecal mucosa [18]. Mixed infections provide a sensitive measure of virulence attenuation referred to as the competitive index [40]. Tenfold serial dilutions of the inoculum were plated to confirm the inoculum titer and input ratio. Fecal pellets were collected daily and cecal contents were collected on day 4 after inoculation for enumerating both WT and *napA* mutant STm CFU. Competitive indices were calculated by dividing the number of *napA* mutant STm by the number of WT STm and normalized to the input ratio.

### RNA isolation and transcript analysis

Ceca were cut open, luminal contents were removed, and tissues were extensively washed with PBS to remove any remaining luminal contents, cut into 0.5 cm^2^ pieces, and transferred into Lysing Matrix D, 2 ml Tubes (MP Biomedicals Cat#6913–100) containing 1 ml TRI Reagent (Molecular Research Center Cat#TR 118). Next, tissues were homogenized for 45 sec using a Mini-Beadbeater-16 (BioSpec Products) and centrifuged to remove debris. RNA was isolated according to the TRI Reagent manufacturer’s protocol. Briefly, chloroform was added to the TRI Reagent suspensions, which were then mixed and centrifuged. The top aqueous layer containing RNA was removed and transferred to a new tube, after which RNA was precipitated with isopropanol. Next, the precipitated RNA was pelleted and washed with ethanol prior to removal of contaminating genomic DNA with TURBO DNA-free Kit (Invitrogen Cat#AM1907), and cDNA was synthesized from the RNA with Verso cDNA Synthesis Kit (Thermo Fisher Scientific Cat#AB1453B). The resulting samples were used as templates for transcript analysis by quantitative PCR. Using gene-specific primers (**S1 Table**) and PerfeCTa SYBR Green FastMix (Quantabio Cat#95072–012), transcript analysis was performed on a CFX96 Touch Real-Time PCR Detection System (Biorad).

### Flow cytometric analysis

Ceca were cut open, luminal contents were collected, and tissues were extensively washed with PBS to remove any remaining luminal contents. Tissues were then cut into 0.5 cm^2^ pieces, washed with PBS, transferred into gentleMACS C Tubes (Miltenyi Biotec Cat#130-096-334) containing 10 ml of dissociation buffer (3 mM CaCl_2_, 5% BSA, and 3U/ml Collagenase Type 2 (Worthington Biochemical Corporation Cat#LS004176) in PBS), twice subjected to gentleMACS Dissociator (Miltenyi Biotec) program *Brain_01*, and incubated for 45 min at 37°C, with agitation. Samples were then twice subjected to gentleMACS Dissociator program *Heart_01* and filtered through a 70 μm nylon filter to obtain single cell suspensions. Cells were then washed twice in PBS and suspended in FACS buffer (1% BSA in PBS) prior to staining. Cells were stained in the presence of Fc block (purified anti-CD16/32 antibody; BioLegend Cat#101302; RRID: AB_312801) with PerCP anti-CD11b antibody (BioLegend Cat#101230; RRID: AB_2129374), APC anti-Ly6C antibody (BioLegend Cat#128016; RRID: AB_1732076), and PE anti-Ly6G antibody (BioLegend Cat#127608; RRID: AB_1186099), and then fixed with Fixation Buffer (BioLegend Cat#420801) according to the manufacturer’s protocols.

To analyze cells present in the cecal lumen, luminal contents were suspended in 5ml PBS, filtered through a 70 μm nylon filter, and washed twice with PBS prior to being suspended in 7 ml of a 30% Percoll solution (100% = 9 part Percoll and 1 part 10x PBS; GE Healthcare Cat#17-5445-02) that was then overlaid onto 4 ml of an 80% Percoll solution and centrifuged for 40 min at 300 x *g*, with the brake off, at room temperature. Cells at the interface between the 30% and 80% Percoll solutions were removed with a Pasteur pipette, added to 10 ml of PBS, pelleted and washed twice with PBS, and then suspended in FACS buffer prior to staining, essentially as above (Zombie Violet Fixable Viability Dye (BioLegend Cat#423114) was added to the staining protocol).

Data were acquired on a BD FACScan flow cytometer (BD Biosciences) with Digital Extra Parameter Upgrade (DxP8) and FlowJo Collectors’ Edition software (both Cytek), and were analyzed with FlowJo v10 software (www.flowjo.com). Where indicated, cells were sorted on a FACSAria flow cytometer (BD Biosciences) in the Stony Brook University Research Flow Cytometry Core Facility.

### Western blot analysis

Ceca were cut open, luminal contents were collected, and tissues were extensively washed with PBS to remove any remaining luminal contents and cut into 0.5 cm^2^ pieces. Next, tissues were transferred into Lysing Matrix D, 2 ml Tubes containing 1 ml RIPA buffer supplemented with cOmplete and PhosSTOP (protease and phosphatase inhibitor cocktails, respectively; Roche Cat#11836153001 and Cat#4906837001), and homogenized for 45 sec using a Mini-Beadbeater-16. Sorted cells were pelleted, washed with PBS, and suspended (2 x 10^7^ cells/ml) in RIPA buffer supplemented with cOmplete and PhosSTOP. Lysates were centrifuged and supernatants were collected. Bradford assay (Bio-Rad Protein Assay Dye Reagent Concentrate; Bio-Rad Laboratories Cat#5000006) was used to determine the protein concentrations of the samples, which were normalized (based on protein concentration), mixed with Laemmli/SDS sample buffer, incubated for 5 min at 95°C, subjected to centrifugation to pellet debris, and loaded onto an 8% Tris-Tricine polyacrylamide gels with discontinuous buffering systems [41]. Following gel electrophoresis, proteins were transferred onto polyvinylidene difluoride membranes by use of a Trans-Blot SD Semi-Dry Transfer Cell System (Bio-Rad).

Expression of iNOS and β-actin was detected with anti-NOS2 antibody (1:1,000; BioLegend Cat#690902; RRID: AB_2629826) and anti-β-actin antibody (1:1,000; Cell Signaling Technology Cat#4967S; RRID: AB_330288), respectively. For detection, Alexa Fluor Plus 800-conjugated goat anti-rabbit IgG secondary antibody (1:10,000; Invitrogen Cat#A32735; RRID: AB_2633284) and Alexa Fluor 680-conjugated goat anti-mouse IgG secondary antibody (1:10,000; Invitrogen Cat#A21058; RRID: AB_2535724) were used in combination with an Odyssey CLx Imaging System (LI-COR). Specific band intensities were quantified, after which relative levels of iNOS were normalized to β-actin.

### Histological analysis

Cecal tips, which contain lymphoid patches, were collected and fixed overnight at 4°C in 10% formalin (Fisher Scientific Cat#SF100-4), after which the samples were extensively washed in PBS before being routinely embedded in paraffin, sectioned (5 μm section per sample), and stained with hematoxylin and eosin in the Stony Brook University Research Histology Core Facility. A board-certified veterinary anatomic pathologist performed blinded, semi-quantitative histopathological analysis of the cecal sections.

### Nitrate measurements

Ceca were harvested, luminal contents were removed, and cecal mucus layers were collected by way of gentle scraping, using the backside of surgical scissors. Cecal mucus was collected in microcentrifuge tubes containing 0.5 ml of sample buffer (Nitric Oxide Assay Kit, Millipore Sigma Cat#482655), after which samples were homogenized by vortexing, centrifuged (10,000 x *g*) for 20 min at 4°C, and filtered using a 10K MWCO centrifugal filter with PES membrane (VWR Cat#82031–348). Levels of nitrate were quantitated using the fluorometric 2,3-diaminonaphtalene (DAN) assay-based Nitric Oxide Assay Kit.

### Quantification and statistical analysis

The significance of the results described in this study was determined by use of a number of different statistical analyses performed with Prism 6 (GraphPad Software; www.graphpad.com). All of the statistical details of the experiments can be found in the figure legends. *P* values of less than 0.05 were considered to be statistically significant. Asterisks indicate statistically significant differences for designated post hoc test comparisons (****, *P* < 0.0001; ***, *P* < 0.001; **, *P* < 0.05; *, *P* < 0.05, n.s., not significant).

## Supporting information

S1 Fig Related to Fig 1. (A) Gating strategy for the flow cytometric analysis performed in this study. (B) Percentage of neutrophils among total gated cells in cecal tissues from C57BL/6J mice (n = 3–6 per group) left untreated or treated with Sm prior to inoculation with PBS or STm, related to Fig 1B and 1C. Ceca were harvested on day 4 after inoculation. Data show mean with SEM and were analyzed by one-way ANOVA with Sidak posttest. (C) Four-day time course tracking neutrophil recruitment into cecal tissues from C57BL/6J mice (n = 4–9 per group) treated with Sm prior to inoculation with STm, related to Fig 1D and 1E. Mice treated with Sm prior to inoculation with PBS (uninfected, UI) were used as a control. Data show mean with SEM and were analyzed by one-way ANOVA with Fisher’s LSD posttest. (D) Percentage of neutrophils among live cells in cecal contents from C57BL/6J mice (n = 6 per group) treated with Sm prior to inoculation with PBS (uninfected, UI) or STm, related to Fig 1F. Cecal contents were collected on day 4 after inoculation. Data show mean with SEM and were analyzed by Student’s *t*-test.
(TIF)

S2 Fig Related to Fig 2. (A) Percentage of neutrophils among total gated cells in cecal tissues from C57BL/6J mice (n = 3 per group) treated with Sm prior to inoculation with PBS (uninfected; UI) or wild-type (WT) STm, or *invA* (T3SS-1), *ssaD* (T3SS-2), or *invA ssaD* (T3SS-1 T3SS-2) mutant STm, related to Fig 2A and 2B. Ceca were harvested on day 4 after inoculation. Data show mean with SEM and were analyzed by one-way ANOVA with Sidak posttest. (B) Percentage of neutrophils among total gated cells in cecal tissues from C57BL/6J (WT), *Ccr2*^-/-^, and *Ccl2*^-/-^ mice (n = 6–9 per group) treated with Sm prior to inoculation with PBS (uninfected, UI) or STm, related to Fig 2C and 2D. Ceca were harvested on day 4 after inoculation. Data show mean with SEM and were analyzed by one-way ANOVA with Sidak posttest.
(TIF)

S3 Fig Related to Fig 4. (A) Level of iNOS expressed in cecal tissues from C57BL/6J (WT) and *Ccr2*^-/-^ mice (n = 6–9 per group) treated with Sm prior to inoculation with PBS (uninfected, UI) or STm, related to Fig 4B. Ceca were harvested on day 4 after inoculation. β-Actin was used as a loading control for Western blotting. (B) Concentration of nitrate in cecal mucus from C57BL/6J (WT) and *Ccr2*^-/-^ mice (n = 4–7 per group) left untreated or treated with Sm prior to inoculation with PBS or STm. Ceca were harvested on day 4 after inoculation. Data show mean with SEM and individual data points, and were analyzed by one-way ANOVA with Sidak posttest.
(TIF)

S1 Table Oligonucleotides. Oligonucleotides used in this study for PCR (*Salmonella* pathogenesis) or quantitative PCR (transcript analysis).
(DOCX)
