# Supplementary figures and images for: Inflammatory monocytes provide a niche for Salmonella expansion in the lumen of the inflamed intestine
Source: PLoS Pathog. 2019 Jul 15;15(7):e1007847. doi: 10.1371/journal.ppat.1007847 (PMC6658010; doi:10.1371/journal.ppat.1007847)

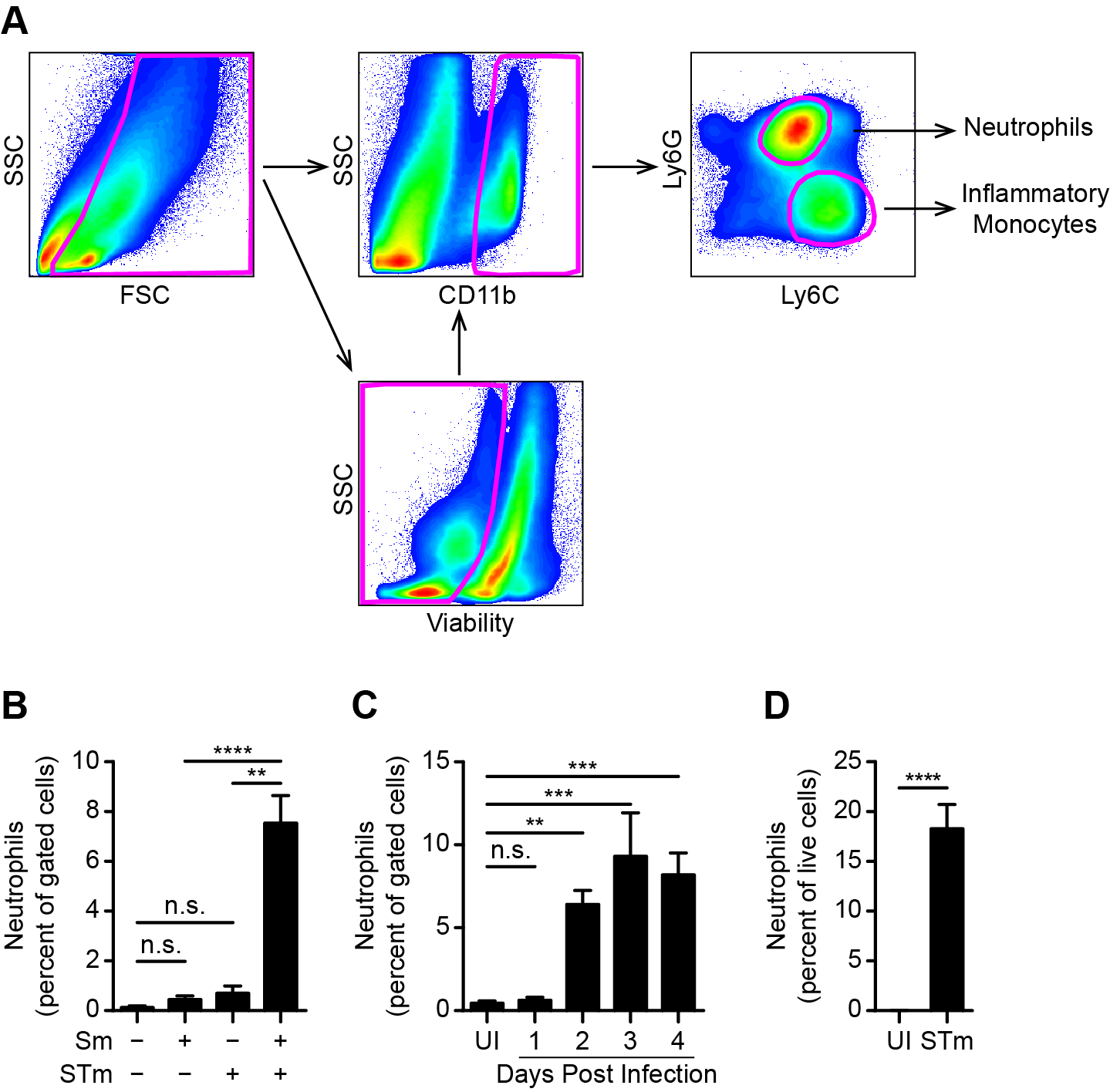

Supplement: S1 Fig — (A) Gating strategy for the flow cytometric analysis performed in this study. (B) Percentage of neutrophils among total gated cells in cecal tissues from C57BL/6J mice (n = 3–6 per group) left untreated or treated with Sm prior to inoculation with PBS or STm, related to Fig 1B and 1C. Ceca were harvested on day 4 after inoculation. Data show mean with SEM and were analyzed by one-way ANOVA with Sidak posttest. (C) Four-day time course tracking neutrophil recruitment into cecal tissues from C57BL/6J mice (n = 4–9 per group) treated with Sm prior to inoculation with STm, related to Fig 1D and 1E. Mice treated with Sm prior to inoculation with PBS (uninfected, UI) were used as a control. Data show mean with SEM and were analyzed by one-way ANOVA with Fisher’s LSD posttest. (D) Percentage of neutrophils among live cells in cecal contents from C57BL/6J mice (n = 6 per group) treated with Sm prior to inoculation with PBS (uninfected, UI) or STm, related to Fig 1F. Cecal contents were collected on day 4 after inoculation. Data show mean with SEM and were analyzed by Student’s t-test. (TIF) [file ppat.1007847.s001.tif]

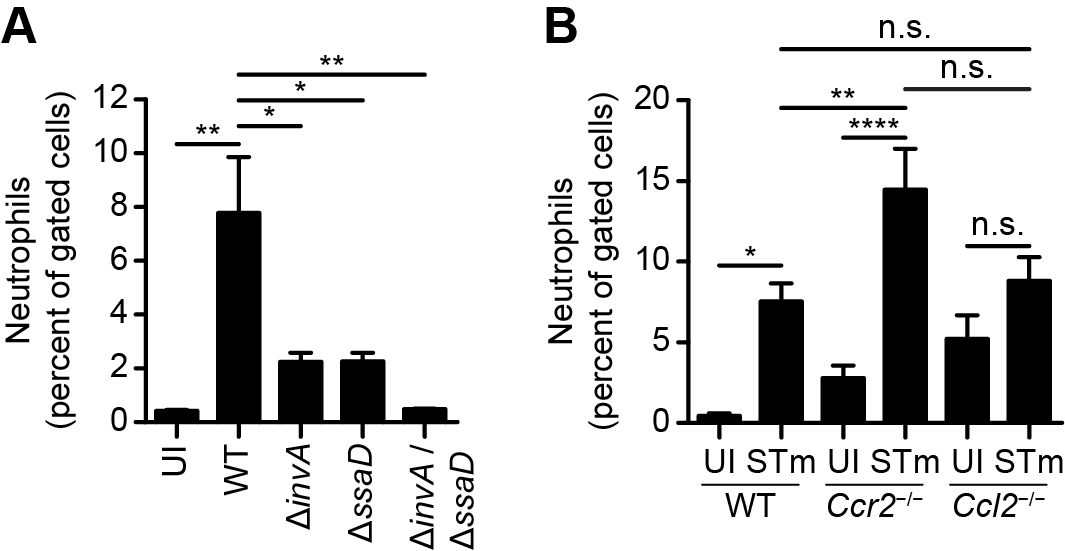

Supplement: S2 Fig — (A) Percentage of neutrophils among total gated cells in cecal tissues from C57BL/6J mice (n = 3 per group) treated with Sm prior to inoculation with PBS (uninfected; UI) or wild-type (WT) STm, or invA (T3SS-1), ssaD (T3SS-2), or invA ssaD (T3SS-1 T3SS-2) mutant STm, related to Fig 2A and 2B. Ceca were harvested on day 4 after inoculation. Data show mean with SEM and were analyzed by one-way ANOVA with Sidak posttest. (B) Percentage of neutrophils among total gated cells in cecal tissues from C57BL/6J (WT), Ccr2-/-, and Ccl2-/- mice (n = 6–9 per group) treated with Sm prior to inoculation with PBS (uninfected, UI) or STm, related to Fig 2C and 2D. Ceca were harvested on day 4 after inoculation. Data show mean with SEM and were analyzed by one-way ANOVA with Sidak posttest. (TIF) [file ppat.1007847.s002.tif]

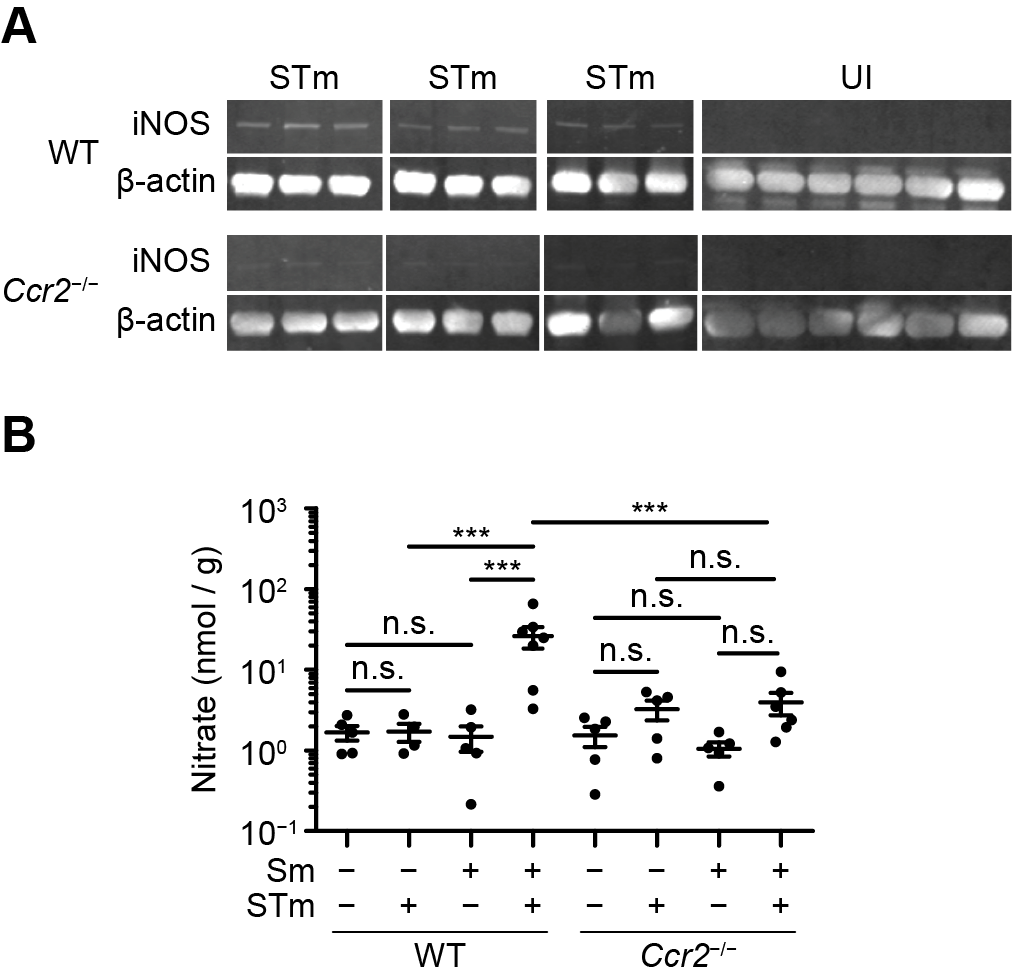

Supplement: S3 Fig — (A) Level of iNOS expressed in cecal tissues from C57BL/6J (WT) and Ccr2-/- mice (n = 6–9 per group) treated with Sm prior to inoculation with PBS (uninfected, UI) or STm, related to Fig 4B. Ceca were harvested on day 4 after inoculation. β-Actin was used as a loading control for Western blotting. (B) Concentration of nitrate in cecal mucus from C57BL/6J (WT) and Ccr2-/- mice (n = 4–7 per group) left untreated or treated with Sm prior to inoculation with PBS or STm. Ceca were harvested on day 4 after inoculation. Data show mean with SEM and individual data points, and were analyzed by one-way ANOVA with Sidak posttest. (TIF) [file ppat.1007847.s003.tif]
