# Supplementary material for: Inflammatory monocytes provide a niche for Salmonella expansion in the lumen of the inflamed intestine
Source: PLoS Pathog. 2019 Jul 15;15(7):e1007847. doi: 10.1371/journal.ppat.1007847 (PMC6658010; doi:10.1371/journal.ppat.1007847)
Supplement: S1 Table — Oligonucleotides used in this study for PCR (Salmonella pathogenesis) or quantitative PCR (transcript analysis). (DOCX) [file ppat.1007847.s004.docx]

**S1 Table. Oligonucleotides.**

| **Primer Name** | **Sequence** |
| --- | --- |
| *Salmonella* Mutagenesis | |
| napA_mut Fwd | TGGTTTATCACCAGCAGGATGAGCAAGGTGAGGAAACACCGTGTAGGCTGGAGCTGCTTC |
| napA_mut Rev | GGCGGCCATTTTGGGGTTTCGCTGTACGGGACATAACGCGATGGGAATTAGCCATGGTCC |
| ssaD_mut Fwd | AGCCTCAGTAGTAAATAATGGCATATCTCATGGTTAATCCGTGTAGGCTGGAGCTGCTTC |
| ssaD_mut Rev | GGTCAAAGTTGTCATTTTCCACTCACTTAAAATCTAATGGATGGGAATTAGCCATGGTCC |
| qPCR | |
| *Ccl2* Fwd | GGCTCAGCCAGATGCAGTTA |
| *Ccl2* Rev | TTCCTTCTTGGGGTCAGCAC |
| *Il1b* Fwd | TACAAGGAGAACCAAGCAACGAC |
| *Il1b* Rev | GCCCATACTTTAGGAAGACACGG |
| *Tnfa* Fwd | CACCACGCTCTTCTGTCTACT |
| *Tnfa* Rev | AACTGATGAGAGGGAGGCCAT |
| *Ifng* Fwd | GGCTGTTTCTGGCTGTTACTG |
| *Ifng* Rev | GGATTTTCATGTCACCATCCTTTTG |
| *Cxcl1* Fwd | ACCGAAGTCATAGCCACACTC |
| *Cxcl1* Rev | CTCCGTTACTTGGGGACACC |
| *Cxcl2* Fwd | AAGTCATAGCCACTCTCAAGGG |
| *Cxcl2* Rev | GGCACATCAGGTACGATCCA |
| *Nos2* Fwd | CAGCTGGGCTGTACAAACCTT |
| *Nos2* Rev | CATTGGAAGTGAAGCGGTTCG |
| *Il22* Fwd | TTGTGCGATCTCTGATGGCTG |
| *Il22* Rev | TCCTTAGCACTGACTCCTCGG |
| *Gapdh* Fwd | TGTAGACCATGTAGTTGAGGTCA |
| *Gapdh* Rev | AGGTCGGTGTGAACGGATTTG |
